# Military service and other socioecological factors influencing weight and health behavior change in overweight and obese Veterans: a qualitative study to inform intervention development within primary care at the United States Veterans Health Administration

**DOI:** 10.1186/s40608-016-0087-3

**Published:** 2016-02-01

**Authors:** Melanie Jay, Katrina F. Mateo, Allison P. Squires, Adina L. Kalet, Scott E. Sherman

**Affiliations:** 1 VHA New York Harbor Healthcare System, 423 East 23rd Street, New York, NY 10010 USA; 2 NYU School of Medicine, 550 1st Avenue, New York, NY 10016 USA; 3 NYU College of Nursing, 285 Mercer St, New York, NY 10003 USA

**Keywords:** Obesity, Primary care, Veterans, Weight management, Focus groups, Qualitative

## Abstract

**Background:**

Obesity affects 37 % of patients at Veterans Health Administration (VHA) medical centers. The VHA offers an intensive weight management program (MOVE!) but less than 10 % of eligible patients ever attend. However, VHA patients see their primary care provider about 3.6 times per year, supporting the development of primary care-based weight management interventions. To address gaps in the literature regarding Veterans’ experiences with weight management and determine whether and how to develop a primary care-based weight management intervention to both improve obesity counseling and increase attendance to MOVE!, we conducted a qualitative study to assess: 1) Veterans’ personal experiences with healthy weight-related behavior change (including barriers and facilitators to behavior change and experiences with primary care providers, staff, and the MOVE! program), and 2) potential new approaches to improve weight management within primary care at the VHA including goal setting and technology.

**Methods:**

Overweight/obese VHA patients (aged 18–75, BMI greater than 30 or greater than 25 with at least 1 co-morbidity) were recruited for focus group sessions stratified by gender, MOVE! referral, and attendance. Each session was facilitated by a trained moderator, audio-recorded, and professionally transcribed. Using an iterative coding approach, two coders separately reviewed and coded transcripts, and met frequently to negotiate codes and synthesize emerging themes.

**Results:**

Of 161 eligible patients, 54 attended one of 6 focus groups (2 female, 4 male, 9–11 participants per session): 63 % were male, 46 % identified as African-American, 32 % White/Caucasian, 74 % were college-educated or higher, and 61 % reported having attended MOVE!. We identified two major themes: Impact of Military Service and Promotion and Sustainability of Healthy Behaviors. After service in a highly structured military environment, Veterans had difficulty maintaining weight on their own. They perceived physical activity as having more impact than diet, but chronic pain was a barrier. We identified individual/interpersonal-, community/environment-, and healthcare system-related factors affecting healthy behaviors. We also received input about Veteran’s preferences and experiences with technology and setting health goals.

**Conclusions:**

Unique factors influence weight management in Veterans. Findings will inform development of a technology-assisted weight management intervention with tailored counseling and goal-setting within primary care at the VHA.

## Background

The United States Preventive Services Task Force recommends that all patients be screened for obesity and offered intensive lifestyle counseling [1] since this can lead to modest weight loss and decreased risk of chronic disease [2, 3]. The prevalence of obesity is 37.4 % in Veteran patients seen at Veterans Health Administration (VHA) medical centers [4] (compared to 34.9 % of the general population [5]), and obesity increases the risk of developing chronic disease [6]. While current VHA resources to address obesity are evidence-based, systematically implemented, and exceed national norms, they still do not adequately address the obesity epidemic in Veterans.

The VHA, the only national single-payer healthcare system in the United States, has over 1700 sites of care and serves close to 9 million Veterans each year [7]. It provides both primary and specialty care within both inpatient and outpatient settings. To combat the challenges of obesity in this population, the VHA requires that Veterans seen within primary care are screened for obesity [6] and that overweight and obese patients are offered intensive treatment through the MOVE! program [8], which is available at all VHA medical centers [9]. Rates of screening and referral to MOVE! and/or offering other treatment options are 94 % due to the use of clinical reminders in the electronic health record [10]. The MOVE! program follows evidence-based obesity treatment guidelines and has a comprehensive, multidisciplinary approach to weight management with group or individual meetings involving discussion, activities, and short lessons to promote lifestyle behavior change. In addition to these in-person options, the program also has options for self-management support via telephone (e.g., TeleMOVE!) [11, 12]. Despite the comprehensiveness of the program, only approximately 19 % of MOVE! participants achieve 5 % weight loss [10, 12]. While this outcome compares favorably to a systematic review and meta-analysis of 68 studies showing that the number needed to treat (NNT) was 5 for 5 % weight loss for behavioral and behavioral plus pharmacologic weight management treatments [13], less than 10 % of eligible patients ever attend one MOVE! session [14, 15]. Potential reasons for poor attendance include barriers related to time, travel and motivation [14, 16, 17]. In addition to lifestyle/behavioral weight management through the MOVE! program, VHA medical centers offer related services such as cognitive behavioral therapy, treatment of disordered eating, diabetes prevention programs, weight maintenance, pharmacotherapy and bariatric surgery depending on location.

The primary care visit is an important time to engage patients in weight management counseling and treatment, and Veterans in the VHA system see a primary care provider 3.6 times per year on average [18]. Primary care at VHA medical centers is organized into multidisciplinary teams, called Patient Aligned Care Teams (PACT), to promote a whole person, team-based approach to care [19]. Every patient is assigned to a “teamlet” consisting of a primary care provider, a registered nurse care manager (with a bachelor’s or associate’s degree), a licensed practical nurse (with a practical nursing certificate), and a clerical assistant. These teamlets work within the larger multidisciplinary team (that includes dietitians, mental health providers, sub-specialists, and healthcare professionals) to coordinate Veterans’ medical, behavioral, and psychosocial health needs.

Even though VHA physicians’ and other providers’ weight management counseling is associated with positive behavioral and weight loss outcomes [6, 20], data from other settings suggests that providers frequently fail to diagnose and counsel obese patients to lose weight [21, 22]. Identified reasons include poor counseling competency [23], competing demands for time during the medical visit [24], and perceived lack of dietary self-control by the patient [25]. VHA providers potentially provide inadequate counseling as well. Only 51 % of obese Veterans have said that they received professional advice to lose weight [26]. This may contribute to low attendance to MOVE!. Thus, effective primary care-based interventions are needed to improve identification and behavioral counseling of obese patients by teamlets to potentially increase MOVE! attendance and provide adequate treatment options for patients who do not wish to participate in intensive programs.

Incorporating goal setting and behavior change technologies into interventions for weight management is supported by the literature, and their potential use in primary care needs to be further explored. Effective weight loss interventions should include the patients’ perspectives, and having them set individualized goals is a strategy supported by many behavior change theories including the Theory of Planned Behavior [27]. A recent systematic review of goal setting for lifestyle behavior change in primary care showed that goal setting is effective in promoting diet and physical activity changes and is associated with weight loss [28–30]. Also, preliminary data from a recent clinical trial using incremental goals to facilitate weight loss demonstrated weight loss 12 weeks post-intervention (−3.8+/−3.6 kg; p = .002) [31–33]. Another systematic review showed that technology-assisted interventions combined with counseling promoted weight loss in primary care settings [34]. Thus, interventions using technology-assisted goal setting have the potential to overcome barriers and facilitate weight management counseling by teamlets. Little is known, however, about Veterans’ experiences with technology and how technology could improve delivery of lifestyle-based weight management services and obesity counseling within primary care at the VHA.

Prior to developing primary care-based weight management interventions at the VHA, we need to understand Veterans’ weight management-related barriers and facilitators, as well as their experiences with weight management counseling within the primary care setting. Unfortunately, few studies have qualitatively examined these issues in Veterans. One study conducted in 2005 found that patients were less likely than primary care providers to perceive that talking to providers about their weight would be helpful and felt that providers blame them for having excess weight [25]. However, this study occurred prior to MOVE! and PACT multidisciplinary team implementation, highlighting the need for more recent and in-depth qualitative data.

In order to address gaps in the literature regarding Veterans’ experiences with weight management and to determine whether and how to develop a primary care-based weight management intervention to both improve obesity counseling and increase attendance to MOVE!, we conducted a qualitative study using focus groups comprised of urban-dwelling Veterans. We aimed to assess: 1) Veterans’ personal experiences with healthy weight –related behavior change (including barriers and facilitators to behavior change and experiences with primary care providers, staff, and the MOVE! program), and 2) potential new approaches to improve weight management within primary care at the VHA including goal setting and technology.

## Methods

We conducted an exploratory qualitative study using focus groups with Veteran patients to closely examine how Veterans communicate their weight management experiences, how they both perceive and receive delivery of obesity counseling and weight management care at the VHA, and what factors impact Veterans’ attitudes and behaviors toward that care. We wanted to explore their attitudes and experiences with regards to goal setting, technology, and the MOVE! program. All research procedures were approved by the Institutional Review Board at the VHA New York Harbor Healthcare System, Manhattan campus.

### Recruitment

**Fig. 1:**
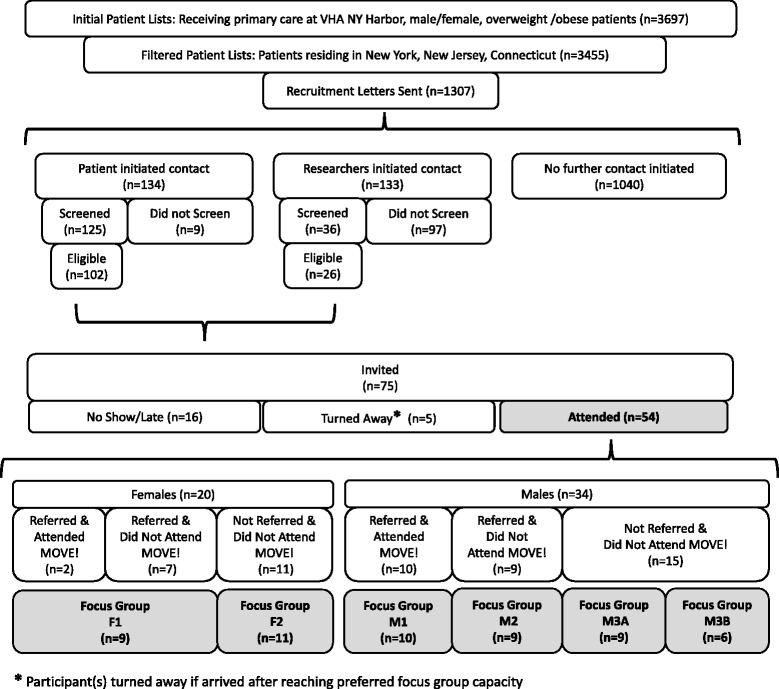
Summary of recruitment and focus group assignment (“F”-females, “M”-males) of Veterans participating in study

Figure 1 summarizes the flowchart of participant recruitment and assignment to focus groups. We identified an initial group of patients from electronic lists compiled by a VHA clinical application coordinator unaffiliated with the study, who extracted patient information based on inclusion criteria. We aimed to recruit patients at the Manhattan VHA medical center for 4–8 focus groups, stopping recruitment once data saturation was achieved [35]. We included Veterans between ages 18 and 75 years old, with a primary care clinic visit within the past 2 years, and a body mass index (BMI) of ≥30 (obese), or BMI ≥25 and <30 (overweight) with at least one comorbidity (hypertension, high cholesterol, type II diabetes, sleep apnea, osteoarthritis, metabolic syndrome). We excluded patients having conditions that interfere with participation in focus groups and/or lifestyle weight management programs. These included (based on ICD 9 codes): organic psychotic condition (e.g., dementia, schizophrenia, major depression, bipolar disorder), psychoactive substance dependence (e.g., alcohol, cocaine, cannabis), chronic rheumatic heart disease, other diseases of endocardium, cardiac dysrhythmia, late effects of cerebral vascular disease, and other cerebral degeneration (e.g., Parkinson’s, mild cognitive impairment). Lists were further filtered to include only patients that reside in New York, New Jersey, or Connecticut for ease of travel.

Recruitment letters were then sent out to a random sample of these patients describing the study, inviting them to contact the research team to participate, and informing them that the research team may call by telephone to recruit. We oversampled female patients because female Veterans only represent approximately 6 % of VHA healthcare users [36], and we wanted to have at least 2 focus groups of only women to get an accurate representation of their views, as well as explore differences between male and female participants. Approximately 20 % of patients who were sent letters either initiated contact or were randomly contacted by the research team (lists of patients were assigned random numbers using a randomized number generator and called in order). Patients were screened by telephone to confirm eligibility requirements and assess basic reading, hearing, and mental capacity to participate in focus groups. For example, we asked patients if they were able to read and understand health forms in English with/without assistance, if they had difficulty hearing or wore a hearing aid, if they had trouble hearing/understanding when several people are speaking in a group, and assessed ease of understanding and communication throughout the recruitment call. Eligible patients were then invited to participate in one of six scheduled focus groups stratified by gender (self-identified as “male” or “female”). As obesity is a stigmatized condition [37], we anticipated that participants would be more comfortable in gender-specific groups [38]. We also attempted to stratify participants by referral/attendance to the MOVE! program according to their electronic medical record.

### Focus group procedures

The investigators developed a semi-structured focus group interview guide for use in Veterans that was informed by the Theory of Planned Behavior [27] and adapted from a version used previously in a separate study of non-VHA Latina primary care patients [39], with additional input from primary care providers and MOVE! staff at the VHA. This guide included open-ended questions to explore the seven domains listed in Table 1.

**Table 1:** Focus group interview guide domains and example questions/probes

Domain	Sample questions
Perceived health risks of obesity	Why do you think health providers (doctors, nurses) advise Veterans like yourselves to lose weight?
Causes of overweight; Barriers to achieving a healthy lifestyle	Why do Veterans like yourselves tend to be overweight?
	(Probes: stress, finances, reasons for poor choices)
Weight loss attempts	Have you ever tried to lose weight? How?
Perceptions of obesity care within VHA primary care	Who at the VHA has discussed your weight with you?
	Who at the VHA should help overweight and obese patients to lose weight?
	How can we better help people to lose weight at the VHA?
Experiences/perceptions of the MOVE! program	Tell me about your experiences with the MOVE! program.
Experiences /perceptions of goal setting	If we talk about goals, what comes to mind?
	What kinds of plans or goals could you make that would help you lose weight?
Experiences/perceptions using technology to assist with healthy lifestyle management	Describe how these devices [smart phones, laptop/desktop computers, tablets, self-monitoring devices] could help you to improve your health.

Focus groups took place in a private conference room at the Manhattan VHA medical center. At first, we invited 10–12 participants per focus group with the expectation that 6–9 would show up. However, attendance rates were higher than expected in the first 3 sessions, which led us to limit the remaining focus group sessions to 9 to maximize both comfort in a small room and participation by all individuals. Spaces were filled on a first come, first serve basis, and if participants completed the session or were turned away before the start of the session, we gave them a $40 cash voucher as compensation for their time. Participants who did not attend or arrived after the focus group session started were not compensated. For each focus group session, we obtained written informed consent to participate and be audio-recorded. Participants then completed a 17-item pre-focus group questionnaire to collect demographic data and obtain information about their weight-loss goals and activities, including participation in the MOVE! program. Each focus group lasted approximately one hour and was led by a trained moderator. Two additional research team members took extensive field notes during each of the focus groups.

### Data analysis

Audio-recordings of each session were transcribed by a professional transcription company. Research team members reviewed transcripts to correct transcription errors and de-identify content. Transcripts were then analyzed using a directed content analysis approach [40]. An initial codebook was developed based on focus group session recollection and field notes. A primary coder first segmented each transcript (where boundaries were mainly determined by individual participant utterances in response to a facilitator question or probe), and with a secondary coder, they both separately reviewed segmented transcripts assigning several codes (up to 7) to each segment. They met frequently to compare coded segments, keeping similar codes and negotiating appropriateness of differing codes (segment codes usually differed by 1–3 codes). They iteratively modified the codebook as analysis proceeded, and new codes emerged until consensus was achieved. The final coded transcripts were imported into NVivo 8 software [41] to assist with data analysis. We used research team discussions, transcript highlights, and field notes to help synthesize themes and define categories. To explore potential differences between groups, we compared code frequency graphs. Emergent themes and related factors were further synthesized and defined, with representative quotes selectively chosen from the transcripts to illustrate themes and categories.

## Results

### Participant characteristics

We screened 161 patients for eligibility, 75 were invited/scheduled to attend, and 54 attended one of six focus groups (see Fig. 1). Table 2 provides a summary of self-reported participant characteristics. The mean age of the participants overall was 58 years (range 27–79), and was lower in the female groups. Participants were predominantly male (63 %), African-American (46 %), completed college or graduate school (74 %), and reported having attended MOVE! (61 %). However, although we attempted to stratify focus groups by MOVE! referral and attendance, the VHA electronic medical record did not accurately capture this information, and some study participants did not recall their prior attendance to MOVE! sessions. The majority of participants had interest in losing weight, and most reported actively trying to lose weight or keep weight off. All participants had a primary care provider and a teamlet and were seen for a wide range of medical and psychiatric conditions. From focus groups discussions, some participants stated that they exclusively received care at the VHA either because they did not have other insurance or because they preferred the VHA over private practitioners. Others said that they saw their VHA provider only to get medications and had another doctor outside the system.

**Table 2:** Participant age, race, ethnicity, education, occupation, MOVE! attendance, and weight loss interest/practice

	Male *n* = 34	Female *n* = 20	Total *n* = 54
Average Age (years)	61	51	58
Race	n (%)	n (%)	n (%)
White	13 (38)	4 (20)	17 (32)
Black	15 (44)	10 (50)	25 (46)
Asian	0 (0)	0 (0)	0 (0)
American Indian	0 (0)	0 (0)	0 (0)
Other^a^	6 (18)	6 (30)	12 (22)
Ethnicity			
Hispanic/Latino	11 (55)	2 (6)	13 (24)
Education Completed			
Less than HS	2 (6)	0 (0)	2 (4)
HS/GED	9 (26)	3 (15)	12 (22)
College	16 (47)	11 (55)	27 (50)
Graduate	7 (21)	6 (30)	13 (24)
Occupation			
Student	2 (6)	1 (5)	3 (6)
Employed	8 (24)	7 (35)	15 (28)
Unemployed	5 (15)	7 (35)	12 (22)
Retired	15 (44)	3 (15)	18 (33)
Other^b^	4 (12)	2 (10)	6 (11)
Attended ≥ 1 MOVE! session	11 (32)	10 (50)	21 (39)
Interested in Losing Weight	30 (88)	19 (95)	49 (91)
Actively trying to lose/maintain weight	23 (68)	15 (75)	38 (70)

^a^Self-reported as biracial or of mixed races, or Hispanic/Latino

^b^Self-reported as disabled, self-employed, or part-time

### Themes

Through thematic analysis, we identified two major themes in relation to weight management for Veterans: Impact of Military Service on Healthy Behaviors (“Military Service”) and Promotion and Sustainability of Healthy Behaviors (“Promotion and Sustainability”). Under Promotion and Sustainability, we organized related factors into 3 major categories corresponding to the socioecological framework [42]: Individual/Interpersonal, Community/Environment, and Healthcare System. To best describe the data, we treated Military Service as both an individual Promotion and Sustainability factor as well as a robust theme unto itself. We describe the themes and categories in more detail below. Of note, when reviewing codes, analyzing language, and conducting our thematic analysis, we did not observe many gender-specific experiences around weight management. Thus, most of the findings described below pertain to both males and females. The few observed gender differences are highlighted in the text.

#### Theme 1: impact of military service on healthy behaviors

We define this theme as factors related to service in the armed forces that impact Veterans’ health and lifestyle choices (see Table 3 for quotes illustrating factors; quotes are referenced here as “3-1”, i.e., Table 3-Quote 1). Participants’ identity as Veterans was very important, considered lifelong, and led to bonding and support in some of the focus groups (3-1). Participants spoke of seeking and enjoying situations where they could give and receive support from each other. Strong military identity and support came up in three of the four male groups. However, the female groups did not express seeking support from other Veterans in particular, and were more likely to speak about doing things on their own or receiving support from friends, family, and healthcare providers.

**Table 3:** Example quotes by focus group (FG) participants related to “Impact of Military Service” theme

Number	Factors	Quote	FG^a^
1	Veteran identity	Here’s the thing about Veterans, is that once you’re one, you’re always one and the bonding that Veterans have is just like…you start telling war stories or back in the day stuff and … it uplifts everybody.	M3B
2	Pressure to maintain weight or stay fit	I was about size 10 and I was a pound overweight and they were gonna put me on the fat girl program, yeah…Well I lost a couple more, just in case, but it does give you discipline to stay in shape… those cockpits are small.	F2
3	Developed poor eating habits	I mean on base we had Burger King and fast-food, and MREs [meals ready to eat] are 3500 cal each, so I mean I continued that once I got out and I’m still eating just as much, but not burning as much so I think our poor eating habits continue.	F2
4	Belief that diet less impactful than physical activity; Chronic pain	Okay, so eating well is a myth to some degree. We know eating well will have an impact, but in terms of my body it doesn’t have a major impact…[If] I can get back into my exercise routine that will probably help me more than anything because I injured my knee so I have stopped jogging. And that’s when I lost control.	M3A
5	Structure in military	But that’s with a lot of soldiers when they get out of the military and no one is tapping you on the shoulder saying, gee, you just gained 20 lb. You should lose it. You don’t. I don’t have to anymore.	M2
6	Lack of structure impacts lifestyle	When you’re not working anymore, and you’re not – you don’t have the structure of going to work every day, you’re not on your feet, moving around, doing things, eating three meals a day, all of sudden…your whole life is unstructured.	M1
7	Lack of autonomy	…sometimes in the military your body and your life aren’t your own. So then when you transition out, all of the sudden it is.	F2
8	Unprepared to make health/life decisions	I mean, when I was in the military I basically had everything taken care of for me because I went in as a kid…you didn’t have to worry about rent, you didn’t have to worry about medical, I mean everything was taken care of for you.	F2
9	Lack of support and services	I’m telling you, you served for your country, in my opinion, they don’t even care. I mean…the Weight Watchers. Why can’t the [VHA] get a discount on that? And the gym…? I mean, there’s so many things that can be helpful for a Veteran.	M2

^a^ *M* male focus group, *F* female focus group

Veterans reported that they were under a lot of pressure while on active military service to maintain an expected weight range and level of fitness, and gaining weight could have impacted their career (3-2). Healthy diets, however, were not emphasized in the military. They had access to fast food restaurants, and there was not an abundance of healthy food choices (3-3). During active service, most participants were significantly younger and more physically fit. Thus, poor eating choices did not cause many of them to gain weight during that time. Participants considered physical activity to be the most important way to lose or maintain a healthy weight and thus continued unhealthy eating habits after leaving service. Even after developing co-morbidities such as diabetes or hypertension, many attributed these to their inability to engage in regular physical activity more than poor diet.

Despite the Veterans’ beliefs that the culture and structure of the military promoted physical fitness and encouraged both males and females to maintain a healthy weight, they found it difficult to sustain these changes once they were discharged. For example, they described chronic back, foot, knee, and shoulder pain from injuries that occurred during their service that interfered with physical activity. While they acknowledged poor eating and medications as causes of weight gain, they most often attributed weight gain to pain and inability to exercise rather than poor food choices (3-4). Individuals who had successfully lost weight usually spoke of doing so by increasing walking, joining a gym, or obtaining exercise equipment.

A lack of daily structure and inadequate life skills were other reasons why participants found it difficult to maintain their weight and fitness levels. In the military, they were required to pass physical tests and experienced structured exercise regiments. Some had been required to wake up early every morning to exercise, and this imposed structure was difficult to maintain after discharge (3-5). In contrast, their current lives were often unstructured especially if they were disabled, retired, or unemployed (3-6). If they worked, they struggled to incorporate physical activity into their lives. While in the military, they had less autonomy and their basic needs were met (3-7). When they were discharged, they often found themselves unprepared to make their own health choices (3-8). Finally, some of the participants expressed frustration that they did not get enough support when they were discharged. Some felt that given the service they gave to their country, the VHA should provide gym memberships and other resources (e.g., pool-based exercises) to help them maintain health (3-9).

#### Theme 2: promotion and sustainability of healthy behaviors

This second major theme was defined as factors that support the initiation, integration, and continuation of healthy behaviors. Quotes in Table 4 illustrate how these factors were discussed by participants and highlight how their experiences were unique to Veterans (quotes are referenced here as “4-1”, i.e., Table 4-Quote 1).

**Table 4:** Example quotes by Focus Group (FG) participants related to “Promotion and Sustainability of Healthy Behaviors” theme

Number	Factor	Quote	FG^a^
		Individual/Interpersonal	
1	Knowledge, Lack of motivation	You know what’s funny, everybody in this room seems to know why it’s important to lose weight…So, it’s one thing to know about something, and something else to act on it and do something about it.	M1
2	Family support	I will be 70 in February. I have an 11-year-old daughter at home. I want to live to at least 100. And I will do whatever I need to do physically to accomplish that.	M3A
3	Emotional eating	…this population is more receptive to stress…they are in conditions from the war where they are disabled or they have other issues and as a result, stress causes you to eat in funny ways, you see? And you eat all the time, you trying to kill the stress.	M3A
4	Personal Finances	When I was on a diet, believe me, I stood broke because if I wanted to eat the proper foods, you have to pay… money flies when you’re on a diet.	M2
5	Goal vs. plan	A goal [is] sort of like the destination…the plan is how you get to that destination.	F2
6	Tracking goals	The goal setting, I just write everything down and check it off. Because when you see it you’re gonna do it.	F1
7	Against goal setting	It’s unobtainable…something you strive for. It becomes then an exercise of frustration and agony and disappointment.	M3A
8	Technology (positive)	Veterans love information so we can set goals, chart it, have it there, constantly input how we’re doing, being able to compare that and see that.	M3B
9	Technology (negative)	Here’s the problem…The internet can be a very dangerous thing. Sure you can find the good information, but you can find some stuff that will absolutely kill you.	M3A
		Community/Environment	
10	Abundance of food availability	In the military, a lot of people didn’t bring big plates of food for everybody. Then you go to work here…I brought a plate of muffins in for everybody and they just set it down.	M2
11	Food hormones	When we grew up there we no such things as human growth hormones and GMOs [genetically modified food] and all of that kind of stuff.	M3A
12	Food advertising	We [are] constantly being bombarded with images of food…we subconsciously believe we won’t be happy unless we have that Big Mac or those fries…	M3B
		Healthcare System	
13	Tailored information	[The dietician] did the portion size, this is what you should eat, this is what you shouldn’t eat, but unfortunately…it wasn’t specifically tailored to me…	F2
14	Research before doctor visit	RESPONDENT 1: I think it is important that all persons that go to the doctor for whatever reason in some, it should be in some form, I can’t say completely educated but have some knowledge about – it is just, if you are going to the doctor about a gallbladder condition…	M3B
		RESPONDENT 2: Thank you. Google it! Google it!	
15	Program feedback	I was recommended to go to the MOVE! program, but after I found out what it was about I didn’t want to do it because I wanted to be on a structured exercise regimen, because I didn’t want to stop eating.	M3A

^a^ *M* male focus group, *F* female focus group

##### Individual/interpersonal factors

Participants described previous successful and unsuccessful weight loss experiences. As described above, most of their attempts had a strong physical activity component (usually walking, jogging, or swimming). They spoke about different ways they tried to change their diet including eating regular meals or pre-packaged meals, limiting food variety, decreasing portions, and juicing.

As is common in many populations struggling with obesity, study participants discussed lack of motivation as a barrier to lifestyle change and weight loss [43]. For this reason, their belief that it was important to maintain a healthy weight often did not translate into ability to sustain healthy behaviors (4-1). For example, several participants described starting programs for weight management, but losing motivation to continue.

For those who had it, support from family and friends was an important facilitator. One female participant spoke about her brother and friends as being important for supporting her rock climbing. Yet another member from the same group responded, “I just want to say that’s good, but I don’t have it [support]. I wish I had it, but I don’t.” Family support was mentioned more often in male groups with discussions about how family members (usually spouses, children, grandchildren) keep them disciplined (4-2). However, others spoke of living alone and being unwilling or unable to cook for themselves.

Barriers to losing weight included high levels of stress and personal issues, often due to their military service, which made them want to eat more than they should (4-3). Participants also indicated that personal finances (4-4) and lack of time prevented them from eating healthy and exercising. Aging and health-related barriers also contributed to weight gain. These included chronic diseases and the need to take medications that caused weight gain, such as insulin for diabetes or steroids for asthma or arthritis. This was especially true among older participants.

We specifically asked participants questions about goal setting as a potential method to help them lose weight. In general, most found goal setting useful and were able to distinguish between a goal and a plan (4-5). Some had received lessons from the MOVE! program or elsewhere about goal setting, and one spontaneously recited the definition of SMART goals [44] that he had learned while in the MOVE! program. Many set behavior change and weight loss goals, while others wanted to improve their physical appearance. Others mentioned non-health or weight-related goals such as getting rich, retiring early, and enjoying new experiences. Some were diligent about writing down and charting their goals (4-6). However, others felt that setting goals could be discouraging if they failed to achieve them. One participant had a particularly negative view of goal setting (4-7).

We also asked about their use of technology in general and, without probing, some participants described using free mobile weight loss applications or “apps” that are commercially available such as My Fitness Pal (http://www.myfitnesspal.com/). These participants spoke about how technology enhances their ability to track their goals (4-8), provides constant feedback (which some said was particularly appealing to Veterans), and gives information about their health without relying on a doctor. However, a few participants in each group were suspicious of technology and had concerns about privacy, security, and misinformation (4-9). Still, others expressed not feeling comfortable using technology in general unless assisted.

##### Community/Local environment factors

Participants spoke about local resources that helped them to lose weight, including low cost public gyms and parks near where they lived. They also spoke about the local food environment as contributing to their difficulty losing weight and making healthy choices. For instance, they cited an over-abundance of food at work and social functions (4-10). They frequented unhealthy restaurants and complained that even the VHA hospital cafeteria served unhealthy food. Many also had the view that the food now commonly contains chemicals/hormones that causes weight gain (4-11). They thought that food advertising also contributed to poor eating habits (4-12).

##### Healthcare and system factors

Overall, participants had mixed views about the care and counseling they received at the VHA. While some complained about access and continuity of care, others had very positive experiences and were impressed by the quality of care they received. When asked about weight management counseling, they reported receiving advice from nurses, doctors, and dietitians, but experienced variability in the quality of counseling. Participants also varied in whom they preferred to deliver the advice. Some felt that doctors should be the ones giving the advice, and some preferred advice from the nurses. Some had received individual counseling by a dietitian, whom they often only saw one time unless they were enrolled in MOVE!. Participants were much more likely to be satisfied with weight management counseling and advice from any healthcare professional if it was individualized and tailored to them (e.g., taking into account their dietary preferences and needs, body type, and physical activity levels) and complained when they perceived the information as generic (4-13). This concept emerged in every group, but more frequently in female groups. Some felt that healthcare professionals provided counseling, “…just so they can have check boxes saying we spoke about nutrition.” They also shared strategies to improve the quality of information that they received from their providers. For instance, some used the internet as a way to research their medical conditions beforehand and/or made lists of questions to ask their doctor so that they could make sure they received the care they needed (4-14).

We asked each group about their perceptions of the VHA’s MOVE! program and found a wide variety of experiences. Some participants did not go to MOVE! because the groups were not scheduled at convenient times, especially if they worked and/or they had to travel far to get to the program location. Cost of travel was an issue for others. Those who had not heard about the program suggested it could be advertised better. Yet, many did not go because it did not offer a structured physical activity component (4-15). Many felt that they already knew what to eat and believed that discussing their diet and health behaviors in a group would not increase their motivation to change. Those who had attended MOVE! specifically enjoyed food demonstrations and the support they received from the group. Some initially lost weight, but gained it back after the program ended. Additionally, a few of the participants had tried the TeleMOVE! program where they were given an electronic scale attached to a messaging device that provided feedback to a case coordinator, as well as a telephone-based education program. Their motivation to participate in TeleMOVE! was time flexibility, and they were drawn to the novelty of new technology.

## Discussion

This qualitative study was designed to assess Veterans’ personal experiences with weight management, particularly within VHA medical centers as well as inform potential ways to improve weight management care at the VHA. While Veterans share many of the barriers to weight loss that impact all populations (e.g., pain and physical activity limitations [45, 46] and stress [47]), we identified two distinct themes describing Veterans’ experiences and beliefs that influence their weight management and lifestyle behavior: 1) Military Service and 2) Promotion and Sustainability of Health Behaviors.

Although it may have been expected that military service would emerge as a theme at a VHA medical center, few studies have reported how it continues to impact Veterans’ lifestyles even years after service. In the military, the lack of autonomy and the highly structured environment may prevent Veterans from learning how to make proper lifestyle choices once they leave service. One study demonstrated similar findings about the role of military service [48]. In interviews with 64 Veterans about their eating behaviors during military service, they found soldiers experienced varying levels of control over food choices and periods of food insecurity while in service that contributed to overeating when they were discharged. They also found high carbohydrate and high fat diets during service contributed to unhealthy habits after service. Indeed, other researchers have documented weight gain after discharge from military service in Veterans from the United States [49] and Belgium [50]. A systematic review found that military deployment impacted body weight and a variety of other health behaviors including excessive drinking and smoking [51].

Several participants in our study believed physical activity was more important than diet for weight loss, even though evidence shows dietary changes drive weight loss more than physical activity [52]. While this belief may be due to lack of knowledge about the importance of diet for weight loss, many of our participants had seen dietitians or had attended the MOVE! program where nutrition education is emphasized. This finding (that many Veterans seem to believe physical activity is central to weight loss) has implications for the delivery of lifestyle interventions in this population. For example, adding more physical activity components (with accommodations and extra support for Veterans with injury or chronic pain) to existing health interventions may increase acceptability to Veterans and encourage them to adopt healthier diets as well. This is supported by cross-sectional studies showing a positive correlation between physical activity and dietary quality [53–55]. Further, even if Veterans do not lose weight with physical activity, it has several health benefits including improved mood [56] and decreased risk of cardiovascular and all-cause mortality [57].

The Promotion and Sustainability theme encompassed several categories including individual/interpersonal and community/environmental factors, as well as interactions with multidisciplinary teams and the larger healthcare system. On an individual level, we found that barriers to weight management included high levels of emotional and financial stress, issues thought to be common in Veterans [58, 59]. Indeed, psychosocial and financial stress are associated with weight gain over time in other populations [60], and this supports using stress management strategies and MOVE! curricula related to stress management to help facilitate weight loss. Most of the participants spoke favorably about goal setting as a strategy for behavior change and weight loss, although they may need more support (e.g., from other Veterans, family, or healthcare providers) to maintain motivation to achieve their goals. On a community level, participants spoke of having access to an abundance of unhealthy, high-caloric food and believed food contained chemicals that caused weight gain. Environmental issues such as these are challenging to address in the clinic setting and highlight the need for partnerships between healthcare providers and community programs. Together, they can work with patients to make small structural changes in their environments to reduce food intake [61]. On a healthcare level, participants wanted weight management advice from healthcare providers at the VHA to be more tailored to their individual needs. Some had not heard of the MOVE! program or had negative impressions.

Even though our focus group guide was informed by the Theory of Planned Behavior [27], some of our findings may fit better with Bronfenbrenner’s Socioecological Model [42]. This model (and its variations) describes how the interplay between the individual and environment impacts human development and behavior and can be used to highlight unique factors that impact the promotion and sustainability of health behaviors [42, 62]. The socioecological model has been applied to explore factors impacting obesity and weight management [63, 64], physical activity interventions [65], as well as risk factors for mental health [66], suicide ideation [67], and PTSD [68] in Veterans. While the socioecological model captures multiple levels of influences on behavior, the constructs of the Theory of Planned Behavior more effectively capture individual/cognitive variables [69]. Attitudes about outcome (e.g., some participants did not believe that attending MOVE! would lead to weight loss), social norms (e.g., in the military, fitness is more important than diet), and perceived behavioral control (e.g., many felt they were unable to lose weight due to injury/inability to exercise) impact Veterans’ intentions to change health behaviors and goals to lose weight. Thus, when designing weight management interventions to improve delivery of care by teamlets and improve attendance to MOVE!, we plan to take both behavior change models into account.

Although several studies show male–female differences in eating patterns (males eat more calories) [70], dieting frequency (females report trying to lose weight more often) [71], weight perception (males are more likely to misperceive their weight status) [72], and weight management program preferences (males may prefer exercise programs) [73], few gender differences emerged in our study. One observed difference was that compared to male participants, female participants sought less social support from other Veterans. We were unable to determine though if the differences we observed reflect age differences (female participants were younger than males) rather than gender. Future research is necessary to explore gender and age differences in weight management preferences and how VHA programs can address them.

This qualitative study has clinical implications that can be used to improve weight management within the VHA healthcare system. The finding that military service potentially impacts obesity and lifelong health behaviors supports the need to improve health behaviors in the military. Indeed in 2013, the Army implemented an initiative called the Human Performance Triad with a focus on improving diet, physical fitness, and sleep in the military [74]. One study that explored the impact of this triad on health and performance showed that soldiers who had healthier diets (as measured by the Healthy eating score) also did better on the Army physical fitness test [55]. Our study suggests that military initiatives such as this may not only increase performance during service, but may also improve Veteran health in the long term.

Veterans want counseling and weight management advice from the health care team to be tailored to their individual preferences and needs. While MOVE! promotes weight loss for Veterans [14] and was attended by over 132,000 patients in 2013 [10], it only reaches a small proportion of the targeted population. VHA initiatives are needed to improve attendance to MOVE!. However, even if such initiatives were to double attendance to MOVE!, services are still needed to provide weight management care for the majority who do not attend. Since Veterans want tailored counseling particularly from their primary care providers, this study supports offering more opportunities for weight management and obesity counseling within teamlets where Veterans are seen frequently [75].

Our findings will inform new interventions to improve primary care-based weight management care at the VHA medical centers. As noted above, goal setting was an acceptable weight management counseling strategy for most participants in our study especially if they obtain support and are held accountable for their goals. Since Veterans in our study have used technology to assist in making health decisions and/or managing their weight, incorporating technology into weight management interventions could be used as one of many strategies to support Veterans and hold them accountable to achieve their goals. Additionally, our findings support ensuring a strong physical activity component and offering services after work hours and remotely. Indeed, several successful programs have been implemented at VHA medical centers to provide remote care via video conferencing and tele-health [76]. Thus, the data from this study will inform the development of a weight management intervention with tailored counseling, goal setting, and integrated technology within primary care at VHA medical centers. Ideally, this intervention will increase attendance to MOVE! and other VHA programs while still providing weight management care to those who do not attend and offer enhanced opportunities for physical activity.

### Limitations

There are several limitations to this study. This was an in-depth study of Veterans at a single facility in an urban setting, and therefore the applicability of the findings to other VHA facilities may vary. Demographically, the majority of the participants had completed college or graduate school, and findings may be different in a less educated group [77]. Unfortunately, we did not collect data about Veterans’ years in service, years since discharge, and starting/ending rank. Thus, we could not evaluate how these variables impacted their weight management experiences. Further, we acknowledge that the impact of military service may have been amplified by the setting. However, the fact that military service figured prominently in all of the groups without specific prompting supports that this theme is robust. Also, we could not evaluate the impact of severe mental health issues such as schizophrenia or bipolar disorder (even though the obesity rates are higher in Veteran populations [78] and psychotropic medications can cause obesity [79]) because we excluded these patients. Similarly, we could not evaluate the impact of substance abuse, another prevalent morbidity factor in this population [80, 81]. While particular highly vocal participants may have biased our findings, we took special care to capture the words of all participants [82]. When voices overlapped, they were difficult to transcribe, a common focus group problem. However, extensive field notes and careful review of all transcripts by research assistants who attended the focus groups limited data loss. Due to limited resources, we could not do a member check with our study population to validate our findings. Finally, since obesity is a stigmatized condition [83], this may have impacted participants’ willingness to disclose various aspects of their experiences.

## Conclusions

To conclude, obesity in Veterans is a complex challenge where the legacies of military service continue to impact lifestyle behaviors post-discharge. Better understanding by health providers of the unique challenges in this group, particularly as they relate to military experience, can help integrate weight management strategies into primary care and more generally at the VHA. As a result, more Veterans may participate in weight management programs and receive obesity-related care, and thus increase the potential for obesity rates to decrease among Veterans.

## Abbreviations

BMI: Body Mass Index
PACT: Patient Aligned Care Teams
VHA: Veterans Health Administration
